# Superior continuous quantity discrimination in a freshwater turtle

**DOI:** 10.1186/s12983-021-00431-y

**Published:** 2021-09-25

**Authors:** Feng-Chun Lin, Martin J. Whiting, Ming-Ying Hsieh, Pei-Jen Lee Shaner, Si-Min Lin

**Affiliations:** 1 School of Life Science, National Taiwan Normal University, Taipei, Taiwan; 2 Department of Biological Sciences, Macquarie University, Sydney, NSW Australia; 3 The Thinking Dog Vet Behaviour Team, Taipei, Taiwan

**Keywords:** Geoemydidae, Learning ability, *Mauremys sinensis*, Reptiles, Weber’s law

## Abstract

**Background:**

Quantity discrimination, the ability to discriminate a magnitude of difference or discrete numerical information, plays a key role in animal behavior. While quantitative ability has been well documented in fishes, birds, mammals, and even in previously unstudied invertebrates and amphibians, it is still poorly understood in reptiles and has never been tested in an aquatic turtle despite the fact that evidence is accumulating that reptiles possess cognitive skills and learning ability. To help address this deficiency in reptiles, we investigated the quantitative ability of an Asian freshwater turtle, *Mauremys sinensis*, using red cubes on a white background in a trained quantity discrimination task. While spontaneous quantity discrimination methods are thought to be more ecologically relevant, training animals on a quantity discrimination task allows more comparability across taxa.

**Results:**

We assessed the turtles’ quantitative performance in a series of tests with increasing quantity ratios and numerosities. Surprisingly, the turtles were able to discriminate quantities of up to 9 versus 10 (ratio = 0.9), which shows a good quantitative ability that is comparable to some endotherms. Our results showed that the turtles’ quantitative performance followed Weber’s law, in which success rate decreased with increasing quantity ratio across a wide range of numerosities. Furthermore, the gradual improvement of their success rate across different experiments and phases suggested that the turtles possess learning ability.

**Conclusions:**

Reptile quantitative ability has long been ignored and therefore is likely under-estimated. More comparative research on numerical cognition across a diversity of species will greatly contribute to a clearer understanding of quantitative ability in animals and whether it has evolved convergently in diverse taxa.

**Supplementary Information:**

The online version contains supplementary material available at 10.1186/s12983-021-00431-y.

## Background

Quantity discrimination, i.e., the ability of animals to discriminate a magnitude of difference (greater vs lesser amounts) or discrete numerical information, plays a key role in their decision-making during foraging (e.g., when to leave a patch), mating (e.g., assessing mate availability), fighting (e.g., assessing the number of opponents), assessing the risk of brood parasitism (e.g. counting eggs in American coots), or predation risk (e.g., schooling in fishes) [1–4]. Quantity discrimination is therefore expected to increase the fitness of animals by elevating their foraging efficiency, breeding success, or survival [1]. Consequently, it is not surprising that researcher have found such a numerical ability in a variety of animal taxa including fishes, birds, and mammals [1, 5], but the mechanism is often unknown [6]. Although discrimination seems to be a common ability present in many animals, it varies across taxa. For example, guppies can discriminate quantities of 4 versus 5 [7], pigeons 6 versus 7 [8], and apes 9 versus 10 [9]. However, studies of numerosity are taxonomically biased, focusing on fishes, birds and mammals. In his review, Agrillo [6] reported that reptiles were the only class of vertebrates for which there were no data on numerosity cognition. He also pointed out that a broader taxonomic coverage is needed to understand the evolution of numerical ability, particularly in understudied groups [6]. In the meanwhile, the gap has been reduced because of new studies on cuttlefish [10], bees [11], spiders [12], salamanders [13, 14], and frogs [15–17]. Growing awareness has led to investigations into the numerical abilities of two lizards [18–20] and a tortoise [21]. This is, however, just a start and far from sufficient for uncovering broad-scale patterns in numerical cognition for such a diverse group as reptiles.

We currently have data on quantity discrimination for only three reptile taxa. Specifically, wall lizards (*Podarcis sicula*) can successfully discriminate between 2 versus 4 [18, 19], gidgee skinks (*Egernia stokesii*) can discriminate quantities up to 3 versus 4 [20], and Hermann’s tortoises (*Testudo hermanni*) can also discriminate 3 versus 4 [21]. In addition to the limited number of reptile species studied, their quantitative ability has not been thoroughly explored. For example, these previous studies stopped testing at small numbers (< 4), hence the ability of reptiles to discriminate large numbers (> 4) remains unknown. Moreover, evidence is growing that reptiles have more sophisticated learning and cognitive abilities than previously assumed [22–24], and even social learning occurs in a non-social tortoise [25]. This suggests that we might have underestimated the quantitative abilities of reptiles for a long time.

There are several primary approaches to test numerosity; two are widely applied: spontaneously measuring quantity discrimination, or using a training method. Spontaneous measures of numerosity have an advantage because they bear more directly on ecological function, such as discriminating food quantity or availability, or the composition of a social group. However, a drawback is that it is difficult to compare across species because the ecological information behind the stimuli are different. For example, Stancher et al. [17] used food as a stimulus to show that Oriental fire-bellied toads (*Bombina orientalis*) can discriminate quantities of 2 versus 3 (a ratio of 0.67) whereas Lucon-Xiccato et al. [16] used grass (potential shelter) as a stimulus to show that Italian treefrogs (*Hyla intermedia*) can discriminate quantities of 1 versus 2 (a ratio of 0.5). In these kinds of studies, Weber’s law is widely applied to evaluate the numerical/quantitative ability of the target species [26]. According to this law, a higher ratio between quantities is numerically/quantitatively more difficult [27, 28]. Hence, one might speculate that the toads have a higher quantitative ability than the frogs. However, given the different contexts in which quantitative abilities were tested, any conclusion on the difference in quantitative abilities between the toads and frogs is at best tentative.

Compared to spontaneously measuring numerosity, training methods use neutral stimuli that provide a baseline to compare cognitive performance across species [29]. Another advantage of training is that it allows for easier control of multiple cues (e.g. when food items were used as stimuli, animals may rely on either visual or olfactory cues, or both). For example, when sunflower seeds were used as stimuli for elephants, seeds were hidden in order to demonstrate that the elephants could use only olfactory cues for quantity discrimination [30]. Compared to the spontaneous method, training tends to be more labor- and time-intensive, and could be constrained by learning effects. However, it provides objective and reproducible information that facilitates cross-species comparisons.

**Fig. 1:**
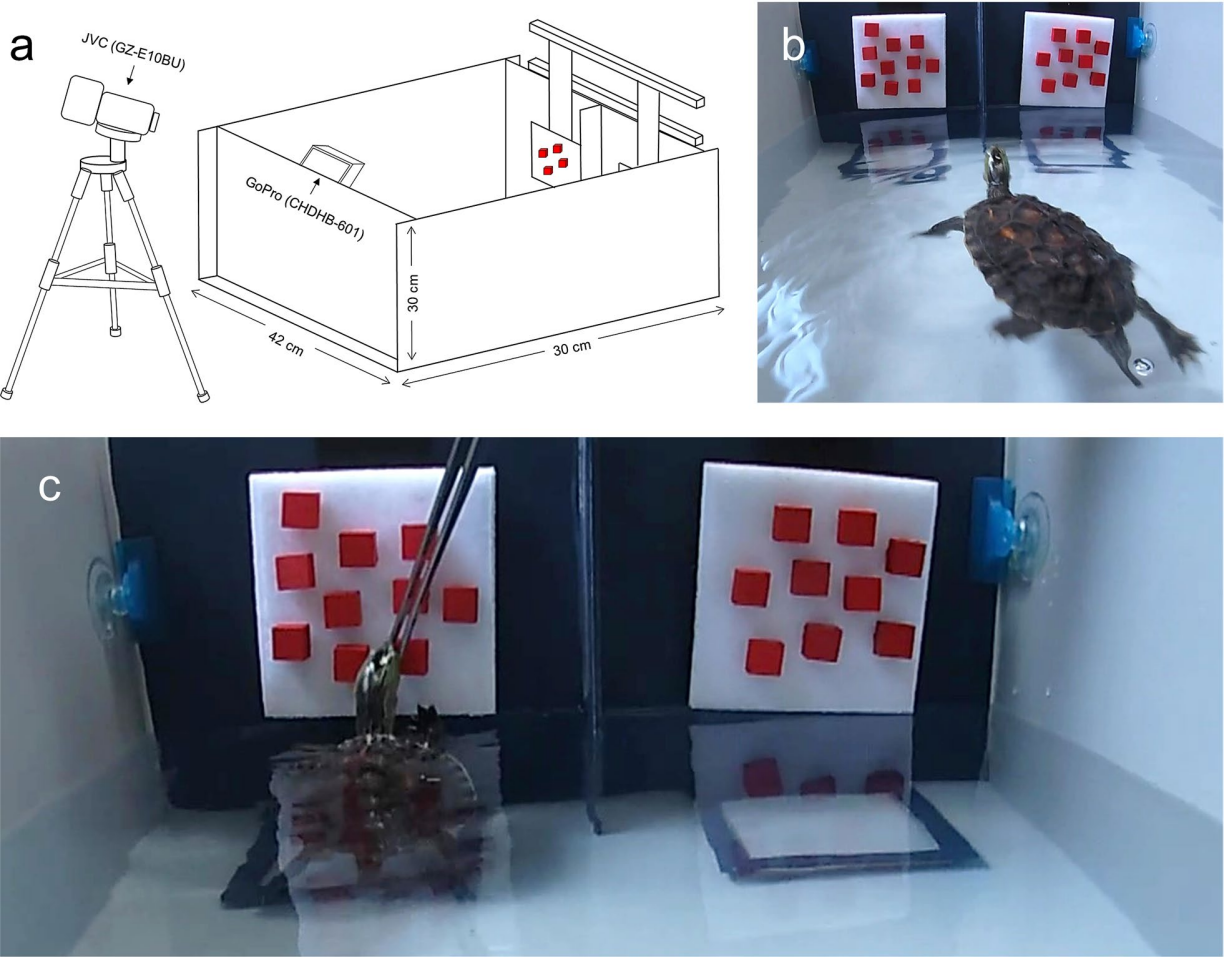
The experiment arena and quantitative stimuli. **A** The experiment arena was an acrylic tank (60 × 42 × 30 cm) filled with water to 15 cm depth. We mounted a GoPro (CHDHB-601) to the back wall of the tank and a JVC camcorder (GZ-E10BU) on a tripod next to the tank. **B** We used wooden cubes (1.5 × 1.5 × 1 cm) colored with red acrylic paints (Mona, SG-203) on a white Velcro board (11 × 11 cm) as the quantitative stimuli. **C** Each turtle was trained to swim toward the stimuli and was rewarded with a food pellet when it reached the designated area (the square marked with blue stripes) for the correct (larger) quantity

Here, we aimed to investigate the quantitative ability of an Asian freshwater turtle, *Mauremys sinensis*, which is one of the most common freshwater turtles in the pet market of East Asia. We trained turtles to discriminate cues that reflected different quantities (Fig. 1 & Additional file 1: Table S1) and tested their ability on a wide range of ratios that increased in difficulty (sequentially increasing ratios and numerosities), which allowed us to test two specific hypotheses: 1) that turtles can learn the concept of “greater than” and improve their ability to discriminate quantity through training and 2) that the quantity discrimination ability of turtles follows Weber’s law.

## Results

### Quantitative ability of the turtles

We trained turtles to establish the “greater than” concept, which was further applied to discriminate unfamiliar quantities. After the training process, five turtles were able to choose the higher quantity from the two stimuli (Fig. 1). In Experiment 1, we conducted fixed numerosity tests, in which a single numerosity pair was repeatedly tested on a turtle before the turtle was switched to a more difficult numerosity pair. In Experiment 2, we conducted mixed numerosity tests, in which multiple numerosity pairs were tested on a turtle within each day. Experiment 2 is much more difficult than Experiment 1 because a turtle had to face at least 10 different numerosity pairings within a single day.

In the fixed numerosity tests (Experiment 1; Additional file 2), the turtles as a group performed significantly better than random (Wilcoxon signed rank tests, *p* < 0.05 for all 5 numerosity pairs; Table 1). At the individual level, they also performed well except for the most difficult numerosity pair (6 versus 7), in which one of the five turtles appeared to be guessing (subject MS31, success rate = 0.59; Table 1). In the mixed numerosity tests (Experiment 2), the turtles as a group generally performed significantly better than random (Wilcoxon signed rank tests, *p* < 0.05 for 30 out of 32 numerosity pairs; Table 1). It is worth noting that the two quantitative tests in which the turtles did not perform well (3 versus 4, 4 versus 5) were an anomaly because they performed well for the most difficult test (9 versus 10; Table 1). Individual turtles were variable in their ability to discriminate quantities. For example, the best performance by MS33 was able to discriminate between 8 and 9. In contrast, MS14 could only discriminate between 6 and 10. Combined, we show that *M. sinensis* as a group could discriminate quantities up to 9 versus 10 with a ratio of 0.9 (Table 1).

**Table 1:** The quantitative ability of Asian freshwater turtles

Pair	Ratio	Difference	Individual level (binominal tests)					Group level (Wilcoxon tests)
			Correct/total trials					
			MS11	MS14	MS16	MS31	MS33	Median success rate
Experiment 1								
1 vs. 3	0.33	2	84/101***	79/100***	77/102***	80/100***	77/100***	0.79*
2 vs. 4	0.50	2	72/100***	68/100***	63/100*	74/100***	80/100***	0.72*
3 vs. 4	0.75	1	68/100***	73/100***	69/100***	74/100***	68/100***	0.69*
4 vs. 5	0.80	1	66/101**	67/100***	71/101***	64/100**	68/100***	0.67*
6 vs. 7	0.85	1	63/100*	64/100**	69/101***	60/101	79/99***	0.64*
Experiment 2 (Phase 1)								
1 vs. 5	0.20	4	7/10	9/10*	10/10**	9/10*	10/10**	0.9*
1 vs. 4	0.25	3	7/10	10/10**	9/10*	9/10*	10/10**	0.9*
1 vs. 3	0.33	2	4/10	10/10**	9/10*	8/10	9/10*	0.9
2 vs. 5	0.40	3	8/10	10/10**	9/10*	7/10	7/10	0.8*
1 vs. 2	0.50	1	8/10	8/10	9/10*	8/10	4/10	0.8
2 vs. 4	0.50	2	7/10	8/10	8/10	5/10	8/10	0.8*
3 vs. 5	0.60	2	6/10	9/10*	8/10	6/10	6/10	0.6*
2 vs. 3	0.67	1	7/10	6/10	6/10	6/10	5/10	0.6*
3 vs. 4	0.75	1	6/10	7/10	5/10	5/10	7/10	0.6
4 vs. 5	0.80	1	6/10	5/10	5/10	7/10	8/10	0.6
Experiment 2 (Phase 2)								
2 vs. 10	0.20	8	9/10*	9/10*	10/10**	9/10*	9/10*	0.9*
2 vs. 8	0.25	6	10/10**	10/10**	10/10**	7/10	10/10**	1.0*
2 vs. 6	0.33	4	5/10	7/10	9/10	7/10	9/10*	0.7*
3 vs. 9	0.33	6	9/10*	10/10**	6/10	7/10	9/10*	0.9*
4 vs. 10	0.40	6	5/10	9/10*	9/10*	10/10**	9/10*	0.9*
4 vs. 8	0.50	4	5/10	8/10	8/10	9/10*	8/10	0.8*
6 vs. 10	0.60	4	7/10	9/10*	9/10*	6/10	6/10	0.7*
4 vs. 6	0.67	2	6/10	8/10	5/10	7/10	6/10	0.6*
6 vs. 9	0.67	3	6/10	7/10	6/10	9/10*	6/10	0.6*
6 vs. 8	0.75	2	6/10	8/10	8/10	8/10	9/10*	0.8*
8 vs. 10	0.80	2	7/10	8/10	8/10	9/10*	6/10	0.8*
Experiment 2 (Phase 3)								
2 vs. 9	0.22	7	9/10*	9/10*	10/10**	10/10**	7/10	0.9*
3 vs. 8	0.38	5	6/10	10/10**	9/10*	10/10**	9/10*	0.9*
4 vs. 9	0.44	5	10/10**	8/10	8/10	10/10**	9/10*	0.9*
3 vs. 6	0.50	3	6/10	9/10*	10/10**	8/10	7/10	0.8*
5 vs. 10	0.50	5	6/10	9/10*	9/10*	10/10**	8/10	0.9*
4 vs. 7	0.57	3	9/10*	7/10	8/10	9/10*	8/10	0.8*
7 vs. 10	0.70	3	6/10	6/10	8/10	7/10	10/10**	0.7*
7 vs. 9	0.78	2	9/10*	6/10	10/10**	7/10	9/10*	0.9*
6 vs. 7	0.86	1	8/10	6/10	6/10	6/10	9/10*	0.6*
8 vs. 9	0.89	1	8/10	6/10	7/10	8/10	10/10**	0.8*
9 vs. 10	0.90	1	6/10	8/10	8/10	8/10	8/10	0.8*

At the individual level, the number of correct choices out of the total number of trials for a given quantitative pair was tested (binomial test) against random (50% correct); at the group level, the success rate (number of correct choices / total number of trials) for a given quantitative pair was tested (Wilcoxon tests) against the median success rate of 0.5. Experiment 1 is the fixed numerosity tests while Experiment 2 is the mixed numerosity tests. The identity of the turtle is indicated by subject ID: MS11, MS14, MS16, MS33 and MS 31. ***p < 0.001; **p < 0.01, *p < 0.05

### Learning ability and ratio dependency in quantity discrimination

**Fig. 2:**
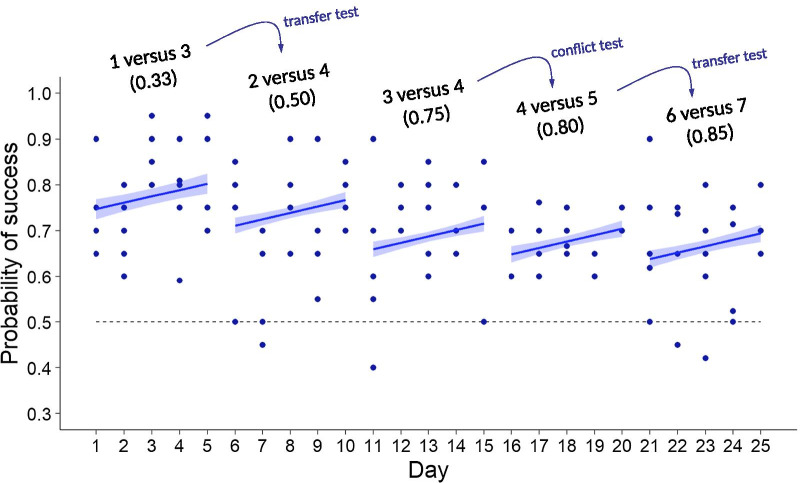
The daily success rate of the turtles in the fixed numerosity tests (Experiment 1). Each numerosity pair was tested 20 times per day on each of the five turtles for five days. The entire experiment lasted 25 days, starting from the lowest ratio (1 versus 3, ratio = 0.33, day 1–5) to the highest ratio (6 versus 7, ratio = 0.85, day 21–25) including two transfer tests and one conflict test. The solid lines with shaded areas are predicted mean probabilities of success ± 1 standard error, estimated from the best-fit model (Table 2 & Additional file 1: Table S2). The horizontal dashed line denotes random choice of the smaller and larger quantity

Learning ability refers to the improvement of performance during the experimental process. In the fixed-numerosity tests (Experiment 1), the daily success rate of the turtles improved over time and decreased with the ratio of the numerosity pair (Fig. 2; Table 2 & Additional file 1: Table S2). The daily success rate among individual turtles (Additional file 1: Table S2) was similar. The performance of individuals improved at a similar rate over the 5-day period, and decreased in a similar way with ratio as well (Additional file 1: Table S2), indicating a lack of individual heterogeneity in learning ability. Absolute difference between the two quantities in a numerosity pair did not affect the turtles’ performance (Additional file 1: Table S2).

**Table 2:** The learning ability, ratio dependency and individual heterogeneity in quantitative discrimination of Asian freshwater turtles

	Estimate	SE	t	*p*
Experiment 1 Best-fit model: Success = Ratio + Day				
(Intercept)	0.80	0.04	21.54	< 0.0001
Ratio	− 0.21	0.05	− 4.47	< 0.0001
Day	0.01	0.01	2.13	0.03
Experiment 2 Best-fit model: Success = Subject + Ratio + Phase				
(Intercept)	0.84	0.04	22.17	< 0.0001
Ratio	− 0.35	0.05	− 6.92	< 0.0001
MS14 (vs. MS11)	0.11	0.03	3.22	0.002
MS16 (vs. MS11)	0.11	0.03	3.22	0.002
MS31 (vs. MS11)	0.08	0.03	2.47	0.01
MS33 (vs. MS11)	0.09	0.03	2.85	0.005
Phase II (vs. I)	0.04	0.03	1.68	0.09
Phase III (vs. I)	0.11	0.03	4.32	< 0.0001

The quantitative discrimination of the turtles was measured as the daily success rate in the fixed numerosity tests (Experiment 1) and 5-day averaged success rate in the mixed numerosity tests (Experiment 2). “Ratio” is the ratio between the two quantities in a numerosity pair; “Day” is the ordered day sequence (i.e. 1, 2, 3, 4, 5) for a given numerosity pair in Experiment 1 indicating continuously accumulated trials; “Phase” is the ordered 5-day sequence (i.e. I, II, III) in Experiment 2 indicating progressively more difficult sets of numerosity pairs; “Subject” is the identity of the turtle. For full model selection details, see Additional file 1: Table S2 & Table S3

### Limits of quantity discrimination and individual heterogeneity

**Fig. 3:**
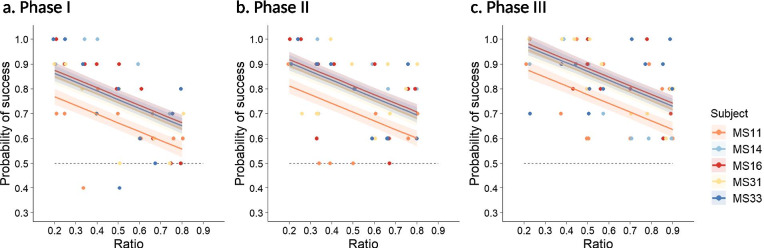
The 5-day averaged success rate of the turtles in the mixed numerosity tests (Experiment 2). Each numerosity pair was tested twice a day in a random order on each of the five turtles for five days. **A** Phase I comprised numerosity pairs of small numbers (1–5) with a ratio ranging 0.2–0.8; **B** Phase II comprised numerosity pairs of at least one large number (6–10) with a ratio ranging 0.2–0.8; **C** Phase III comprised numerosity pairs of at least one large number (6–10) with a ratio ranging 0.2–0.9. The solid lines with shaded areas are predicted mean probabilities of success ± 1 standard error, estimated from the best-fit model (Table 2 & Additional file 1: Table S3). The dots are the observed success rates with 10% jittering using jitter function in R v3.6.1, and the horizontal dashed line denotes random choice of the smaller and larger quantity

In the mixed numerosity tests (Experiment 2; Additional file 2), the individuals also improved across the three phases and decreased performance with increasing ratio of the numerosity pair, and differed among individual turtles (Fig. 3; Table 2 & Additional file 1: Table S3). The performance of the individuals improved across the three phases at a similar rate and decreased with bigger ratios in a similar way too (Additional file 1: Table S3). The difference among individuals was due to one single underperformer (MS11; Fig. 3), which performed worse in the more complicated Experiment 2 but not in the simpler Experiment 1. These patterns suggest that individual heterogeneity might become evident when the turtles approached their limits in quantitative ability, or when the tasks were so complicated that the learning effect could not provide a significant contribution without a large number of repetitions (such as in Experiment 1). Absolute difference between the two quantities in a numerosity pair did not affect the turtles’ performance (Additional file 1: Table S3).

## Discussion

### Quantity discrimination in turtles

*Mauremys sinensis* can successfully discriminate quantities of up to 9 versus 10 (ratio: 0.9), which has seldom been shown in ectotherms. Based on currently available data, the ability of *M. sinensis* to discriminate quantities appears to be superior to guppies (4 versus 5, [7]), lizards (2 versus 3 [18] or 3 versus 4 [20]), and pigeons (6 versus 7, [8]). However, because animals tend to perform better if both discrete (number) and continuous (size, area, circumference) information are available [31], we caution that the seemingly superior quantity discrimination of this turtle could be a result of having both discrete (number) and continuous (size, area, circumference) information available. Therefore, to rigorously compare across species from different studies, the type of stimuli and numerical information available (e.g., whether or not continuous properties are controlled) must be explicitly considered. Our findings, however, highlights that numerosity and other cognition abilities in turtles and other reptiles has likely been long underestimated.

One of the reasons that we were able to demonstrate superior quantity discrimination ability in this turtle is because we used extensive training with an intensive schedule. For example, Bissazza et al. (2014) [7] reported that guppies were able to discriminate quantities up to 4 versus 5 with 120 training trials, better than the closely related mosquitofish (2 versus 3, [32]) that experienced fewer training trials. In another study, DeLong et al. [33] provided goldfish with 1,200 training trials and reported that their success rate was 91% after 450 trials. This performance was higher than previous studies on fishes with fewer training trials (correct rates 55–75%, [7, 32]). In our study, each turtle was subject to around 820 trials (500 trials in Experiment 1, and 320 in Experiment 2). Although the total number of trials was relatively low compared to the thousands of trials reported in some mammal and bird studies [33–36], we conducted these trials within a short time-frame. The extensive training with the intensive schedule is in sharp contrast to most studies on ectotherms where subjects were trained either with a lower intensity or with only a few trials/day [7, 18]. The training method we employed, along with the availability of both discrete and continuous information, likely helped the turtles to approach their limits in quantitative ability. Finally, their body size, coupled with a hearty appetite, helped make them excellent subjects for our experiments. Every morning during the testing period, all the turtles showed a strong urgency to enter the experimental arena, suggesting that food-based training was particularly effective for testing discrimination ability.

### Quantity discrimination follows Weber’s law

Our results strongly support the prediction from Weber’s law. As ratios increased (0.2–0.9), the turtles’ quantitative performance decreased across 32 numerosity pairs that represented various combinations of small and large numbers. This ratio dependency in quantity discrimination was similar among individuals and was not altered through learning. Our finding adds a strong case to the growing evidence that Weber’s law applies to all major vertebrate groups, including fishes [37], amphibians [3], reptiles [21], birds [38], and mammals [39].

### Learning ability of turtles

To the best of our knowledge, this is the first report that demonstrates a turtles’ ability to establish an abstract quantitative concept through learning. Our experiment contained numerous “transfer” tests and “conflict” tests [11], which are difficult to process unless the turtles had truly established the “greater than” concept. Transfer tests refer to when a turtle was challenged by new quantities that they had never encountered before, such as the process from 1 versus 3 to 2 versus 4 (Fig. 2). Conflict tests refer to when a turtle had to give up a previously chosen quantity (e.g., to choose 4 as the larger quantity in the 3 versus 4 pair) and pick another in order to be successful (to abandon 4 and choose 5 in the 4 versus 5 pair) (Fig. 2). The turtles showed not only the ability to learn quantity discrimination, but also the ability to learn as they continued improving the success rate over the experimental period. From the beginning of Experiment 2, they began to handle both transfer and conflict tests within a short period which appeared repetitively as their daily tasks.

The learning effect could also be seen by comparing the performance of the turtles on the more difficult tests between Experiment 1 and 2. Specifically for 3 versus 4 and 4 versus 5, which were included in both Experiment 1 and 2. The turtles performed better in Experiment 1 than Experiment 2, suggesting that the high number of trials and gradual progression from easy to difficult ratios in Experiment 1 might have helped tune their visual perceptual system for better performance, which probably could not be achieved with the low number of trials and mixed ratios in Experiment 2.

One of the five turtles (MS11) became an underperformer in the later stages (Experiment 2) despite doing just as well as the others in the earlier stages (Experiment 1). Such individual heterogeneity might be related to the increasing numerosity challenges and/or lower intensity of training in Experiment 2, personality (e.g. bolder individuals may learn faster but reach limits earlier), or combinations of these factors. Future studies that implement extensive training across all numerosity challenges, or simultaneously track individual differences in quantitative/numerical performance and personality, could greatly contribute to our understanding of the interplay between behavior and cognition [40].

### Implications and future directions

We readily acknowledge that more explicit methods should be applied to prevent our results from confounding with the continuous properties of our stimuli, such as the total surface, convex hull, and density, which correlated with quantities. Based on the current experimental design, we could not determine whether the turtles made their judgements by the quantity or by these continuous properties. The main deficiency of this design was the positive correlation between the quantity and the total surface area or convex hull of our stimuli, because the size of the cubes is identical, and the inter-distance between nearby cubes were even. Therefore, the turtles might be able to make the judgements using "size perception" by integrating all the cubes as a "single object" [41]. We suggest that future studies should: 1) vary the size of each object to prevent turtles from using continuous properties; 2) vary the shape of the object to confirm their use of the “greater than” concept; or 3) attempt to use the delayed-matched-to-sample paradigm, which might be a more explicit method to present the conflict test and transfer test.

Furthermore, relatively little is known about the adaptive value of a quantitative ability in turtles. For example, male guppies could assess operational sex ratio of shoals and joined the one with more females to increase their reproductive opportunities [1]. American coots use enumeration and memory to recognize their own eggs to prevent intra-specific brood parasitism [2]. Group living animals ranging from ants to chimpanzees use quantitative ability to judge opponent groups when in conflict [42, 43]. Yet, there have been few studies on the ecological functions or fitness benefits of quantitative ability in turtles. Together, this study revealed high-level cognitive ability of the Asian freshwater turtle, offering a new system for which the role of cognition in ecology and evolution can be explored.

## Conclusions

Our study revealed a high proficiency in quantity discrimination ability of a reptile, which suggests that the cognitive ability of reptiles has long been underestimated. We further demonstrated that using the training method has the potential to reveal the upper limits of reptilian quantitative ability as well as individual variation in ability as they reach such limits. This experimental system provides new opportunities to explore the link between cognitive performance and fitness of turtles, which could be acquired from long-term studies in the wild or through behavioral experiments. Future studies on numerical cognition in reptiles and other underrepresented animal taxa will help lay the foundation for a comprehensive understanding of animal quantity discrimination and the evolution of numerical ability.

## Materials and methods

### Experimental design

We trained the turtles to apply the “greater than” concept, which was further applied to discriminate unfamiliar quantities. The aim of the training was to test the limits of the turtles’ quantitative discrimination ability in a dichotomous choice task, and within-subjects design. In Experiment 1, we provided fixed numerosity tests with increasing difficulty according to Weber’s law. A total of five quantitative pairs, up to 6 versus 7, were sequentially conducted, with each pair tested over five consecutive days. In Experiment 2, we conducted mixed numerosity tests, which included 10 to 11 different quantitative pairs. A total of 32 pairs, comprising small numbers and large numbers up to 9 versus 10, were tested from three phases with gradually increasing difficulty.

### Experimental arena and quantitative stimuli

The experiment arena is detailed in Fig. 1A. We presented a choice between red-colored wooden cubes (1.5 × 1.5 × 1 cm) on white Velcro boards (11 × 11 cm) (Fig. 1B). The number of the cubes was the quantitative stimulus; each turtle was pre-trained to swim to a designated point marked with blue stripes directly underneath the chosen stimulus (see details in *Subject and training*; Fig. 1C). In each trial, the two stimuli (the white boards with red cubes) were simultaneously presented to the turtle, and immediately removed after the choice had been made. The cubes were arbitrarily placed to form various shapes and arrangements across trials, which was designed to lower the chance of the turtles using geometric pattern or density as a cue. We assigned the side with the higher quantity following a modified pseudorandom Gellermann sequence [44] in which the higher quantity would not appear on the same side more than twice in a row (Additional file 1: Table S4) to control for a potential side bias.

### Subjects and pre-training

The stripe-necked turtle (Geoemydidae: *Mauremys sinensis*) is one of the most abundant captive-bred freshwater turtles in East Asia. Fourteen juveniles, plastron length 8–10 cm, were acquired from a licensed turtle farm in the autumn of 2018. The turtles were kept in a fenced indoor space (200 × 160 cm) containing a rectangular pool (120 × 80 × 30 cm). The ambient temperature was kept at 26 ℃ to conduct the experiments, with natural light. Turtles were fed every other day with commercial food pellets, which were also used as rewards throughout the entire experiment. All the procedures used in this study followed protocols approved by Institutional Animal Care and Use Committee (IACUC) of National Taiwan Normal University (license No. 107029).

The pre-training began in April 2019 and involved four steps: 1) learning to acquire food pellets from a pair of tweezers; 2) learning the association between the quantitative stimulus (three red cubes on a white board) and food rewards; 3) learning to choose the higher quantity from the two stimuli (rewards were given only when the higher quantity was chosen); and 4) learning the association among the designated area, quantitative stimulus, and food rewards (Fig. 1). Correct behaviors were reinforced with food rewards and no negative reinforcement was used. Seven among the fourteen turtles, with higher boldness and better appetite, passed the tweezers training (the first step) faster than the others and were selected to continue the process. Five of the seven turtles passed all the remaining three steps and went on to the testing phases.

### Experiment 1: Fixed numerosity tests

Experiment 1 was designed to test whether the turtles can learn to discriminate quantities and whether their discrimination ability is ratio dependent as predicted by Weber’s law (animals’ discrimination ability decreases with increasing ratios between quantities). Within the 25-day schedule (April 29th–May 23th 2019), five fixed numerosity tests of different ratios were applied sequentially, starting with the easiest numerosity pair 1 versus 3 (ratio = 0.33), and progressing to 2 versus 4 (0.50), 3 versus 4 (0.75), 4 versus 5 (0.80) and 6 versus 7 (0.86). Each of the five turtles received a fixed numerosity test of a given ratio for a total of 99–102 trials (around 20 trials per day for five consecutive days), before moving on to the next numerosity test of another ratio (Fig. 2). In order to move to the next ratio, a turtle had to make a significantly correct proportion of choices; i.e. nonrandom choices (Wilcoxon signed rank test). All turtles passed the five numerosity tests (one individual, MS31, had a marginally significant success rate in the most difficult numerosity pair 6 versus 7; Table 1).

### Experiment 2: Mixed numerosity tests

Experiment 2 was designed to explore the limits of the turtles’ ability to discriminate quantity by using a wide range of numerosity combinations (small and large numbers) and ratios (0.2–0.9). Since there was an almost one-month gap, a five-day training, with mixed numerosity pairings of 20 trials per day, was conducted between 21–25 June to reinforce what had been learned by the turtles during Experiment 1 (the last day of Experiment 1 was 23 May). Experiment 2 was then conducted continuously from 26 June to 11 July, with the exception of a one-day break between Phase II and Phase III (Additional file 2).

The difficulties of the quantitative combinations were gradually increased in three phases. In Phase I, the numerosity pairs were a combination of small numbers (1–5) with a range of ratio 0.2–0.8. In Phase II, the numerosity pairs contained at least one large number (6–10) with a range of ratios 0.2–0.8, such as 6 versus 8, 6 versus 9, and 8 versus 10. In Phase III, the numerosity pairs also contained at least one large number (6–10) as in Phase II, but the range of ratios was further extended to 0.9 (e.g., 7 versus 9, 8 versus 9, and 9 versus 10). Each phase lasted 5 (consecutive) days, and on each day, a turtle received 2 trials for a given numerosity pair with a total of 20–22 trials covering multiple quantitative pairs (hence “mixed” numerosity tests). A turtle received 10 trials for any given numerosity pair over the 5-day period in Experiment 2. All other procedures were the same as in Experiment 1 (Additional file 1: Table S2).

### Data collection

FCL conducted Experiment 1 by herself continuously for 25 days. In order to rule out the “Clever Hans effect”, FCL and SML conducted Experiment 2 together. While a turtle was swimming back to the start point, SML changed the quantities and arrangements of the cubes. Before the turtle made the choice, FCL did not know which side of numerosity was higher until she was informed by SML. After receiving a signal from SML, FCL conduced the treatments: providing a food pellet as reward when the choice was correct, or removing the stimuli (no reward) when the choice was false.

All trials were recorded by a GoPro (CHDHB-601) on the back wall of the tank and a JVC camcorder (GZ-E10BU). FCL watched all the videos and scored the dichotomous outcome of each trial, either “correct (choosing the higher numerosity)” or “incorrect (choosing the lower numerosity)” in each turtle, which was double-checked by another technician (Pin Xuan Lim).

### Statistical analyses

To test whether the quantitative performance of the turtles was better than random, we performed binomial tests at the individual level (null hypothesis: number of correct choice > 50% of total number of trials) and Wilcoxon signed rank tests at group level (null hypothesis: median success rate > 0.5; success rate is the number of correct choice / total number of trials).

For Experiment 1, we fitted linear models to the daily success rate (number of correct choice / number of trials per day), with subject (turtle identity), ratio (ratio of the two quantities in a pair), day (day 1 through 5) and their interactions as fixed effects. The residuals of the full model met the assumption of normality. Because it is not possible to create various numerosity combinations without changing the absolute difference between the two quantities in a pair, we considered the difference as a covariate in the model. We did not incorporate the difference as a fixed effect because we had only two levels of differences in this experiment and they were very close (1 and 2; Table 1). We examined the effect of the quantity difference explicitly in Experiment 2 (see below). We started with the full model (daily success rate = subject + ratio + day + subject × ratio + subject × day + ratio × day + subject × ratio × day + difference) and reduced it to the best-fit model for parameter estimates through model selection based on likelihood ratio tests (LRT). Model selection details can be found in Additional file 1: Table S2.

For Experiment 2 (Phases I, II, and III), we fitted linear models to the success rate (number of correct choice / number of trials over the 5-day period), with subject (turtle identity), ratio (ratio of the two quantities in a pair), difference (absolute difference between the two quantities in a pair), and their interactions as fixed effects. The interactions involving both ratio and difference were excluded from the full model because ratio and difference are highly correlated (r =  − 0.76, *p* < 0.0001). Because the turtles might continuously improve their ability through phases, we considered the phase (I, II, III) as a covariate in the models. We started with the full model (success rate = subject + ratio + difference + subject × ratio + subject × difference + phase) and reduced it to the best-fit model for parameter estimates through model selection based on LRT. Model selection details are in Additional file 1: Table S3.

All statistics were performed in R v3.6.1.

## Supplementary Information

**Additional file 1: Table S1**. A list of sample videos in each experimental stage; **Table S2**. The pseudo-randomized sequence of tests applied in Experiment 2; **Table S3**. Model selection for the quantitative ability of Asian freshwater turtles in Experiment 1; **Table S4**. Model selection for the quantitative ability of Asian freshwater turtles in Experiment 2.

**Additional file 2**. Data of daily performance of each turtle in each experiment.

## Data Availability

All the data has been provided in the supplementary information which accompanies this paper.
