# Supplementary material for: Superior continuous quantity discrimination in a freshwater turtle
Source: Front Zool. 2021 Sep 25;18:49. doi: 10.1186/s12983-021-00431-y (PMC8466656; doi:10.1186/s12983-021-00431-y)
Supplement: Supplementary file 1 — Additional file 1: Table S1. A list of sample videos in each experimental stage; Table S2. The pseudo-randomized sequence of tests applied in Experiment 2; Table S3. Model selection for the quantitative ability of Asian freshwater turtles in Experiment 1; Table S4. Model selection for the quantitative ability of Asian freshwater turtles in Experiment 2. [file 12983_2021_431_MOESM1_ESM.docx]

**Supplementary Information**

**Superior continuous quantity discrimination in a freshwater turtle**

Feng-Chun LIN^1^, Martin J. WHITING^2^, Ming-Ying HSIEH^3^,

Pei-Jen Lee SHANER^1,*^, Si-Min LIN^1,*^

^1^ School of Life Science, National Taiwan Normal University, Taipei, Taiwan

^2^ Department of Biological Sciences, Macquarie University, Sydney, Australia

^3^ The Thinking Dog Vet Behaviour Team, Taipei, Taiwan

*Corresponding Authors

Pei-Jen Lee SHANER

Email: [pshaner@ntnu.edu.tw](mailto:pshaner@ntnu.edu.tw)

Tel: +886-2-77496301

ORCID ID: 0000-0001-8112-4299

Address: No. 88, Tingzhou Rd. Sec. 4, Taipei 116, Taiwan

Si-Min LIN

Email: [lizard.dna@gmail.com](mailto:lizard.dna@gmail.com)

Tel: +886-2-77496246

ORCID ID: 0000-0001-7080-706X

Address: No. 88, Tingzhou Rd. Sec. 4, Taipei 116, Taiwan

**Supplementary Table S1.** A list of sample videos in each experiment stage.

| Experiment | Numerical test | Turtle | Success rate | Video link |
| --- | --- | --- | --- | --- |
| 1 (fixed numerosity) | 1 versus 3 (0.33) | MS11 | 95% | <https://youtu.be/qM-Ru2DuPx8> |
|  | 2 versus 4 (0.50) | MS33 | 90% | <https://youtu.be/uWpsP4VF7Sw> |
|  | 3 versus 4 (0.75) | MS31 | 90% | <https://youtu.be/XAJSTDVY2iA> |
|  | 4 versus 5 (0.80) | MS33 | 75% | <https://youtu.be/KjxTBP3m1a4> |
|  | 6 versus 7 (0.85) | MS33 | 90% | <https://youtu.be/6CXN3U88DK4> |
| 2 (mixed numerosity) | Phase I | MS14 | 85% | <https://youtu.be/YpQ34l43b-I> |
|  | small numbers 1-5, ratio 0.2-0.8 | MS16 | 80% | <https://youtu.be/OpjikOD6_bQ> |
|  | Phase II | MS14 | 91% | <https://youtu.be/C81k50C9aE4> |
|  | large numbers 6-10, ratio 0.2-0.8 | MS33 | 91% | <https://youtu.be/4QzVMII5KtU> |
|  | Phase III | MS31 | 91% | <https://youtu.be/smK9aTy026U> |
|  | large numbers 6-10, ratio 0.2-0.9 | MS33 | 91% | <https://youtu.be/vhQV6dr3PNE> |
| Full version |  |  |  | <https://reurl.cc/j8EQNM> |
| Essence version |  |  |  | <https://youtu.be/XvpYcOaTYyM> |

**Supplementary Table S2.** The pseudo-randomized sequence of tests applied in Experiment 2

Phase I

| Tests | Day 1 | Day 2 | Day 3 | Day 4 | Day 5 |
| --- | --- | --- | --- | --- | --- |
| 1 | 2 vs 4 | 1 vs 3 | 2 vs 1 | 1 vs 4 | 4 vs 1 |
| 2 | 4 vs 1 | 4 vs 2 | 2 vs 5 | 4 vs 5 | 4 vs 2 |
| 3 | 5 vs 2 | 2 vs 5 | 4 vs 5 | 4 vs 2 | 3 vs 5 |
| 4 | 3 vs 5 | 5 vs 3 | 3 vs 2 | 3 vs 5 | 4 vs 3 |
| 5 | 3 vs 4 | 4 vs 3 | 3 vs 5 | 5 vs 2 | 2 vs 3 |
| 6 | 3 vs 1 | 1 vs 2 | 3 vs 1 | 5 vs 1 | 4 vs 5 |
| 7 | 2 vs 3 | 4 vs 5 | 4 vs 3 | 3 vs 4 | 5 vs 2 |
| 8 | 1 vs 2 | 4 vs 1 | 1 vs 5 | 3 vs 1 | 1 vs 5 |
| 9 | 5 vs 1 | 2 vs 3 | 4 vs 2 | 1 vs 2 | 1 vs 2 |
| 10 | 5 vs 4 | 5 vs 1 | 1 vs 4 | 3 vs 2 | 3 vs 1 |
| 11 | 2 vs 4 | 3 vs 2 | 1 vs 2 | 1 vs 4 | 1 vs 2 |
| 12 | 5 vs 1 | 1 vs 5 | 5 vs 3 | 5 vs 1 | 5 vs 3 |
| 13 | 4 vs 3 | 1 vs 4 | 5 vs 1 | 3 vs 2 | 2 vs 4 |
| 14 | 3 vs 5 | 5 vs 3 | 1 vs 3 | 1 vs 3 | 5 vs 1 |
| 15 | 5 vs 4 | 2 vs 4 | 4 vs 2 | 5 vs 4 | 4 vs 3 |
| 16 | 3 vs 1 | 1 vs 2 | 4 vs 3 | 2 vs 5 | 2 vs 5 |
| 17 | 1 vs 4 | 4 vs 3 | 2 vs 3 | 1 vs 2 | 3 vs 1 |
| 18 | 2 vs 3 | 5 vs 4 | 4 vs 1 | 4 vs 3 | 4 vs 5 |
| 19 | 2 vs 1 | 2 vs 5 | 4 vs 5 | 2 vs 4 | 4 vs 1 |
| 20 | 2 vs 5 | 3 vs 1 | 2 vs 5 | 5 vs 3 | 2 vs 3 |

**Supplementary Table S2 (continued).**

Phase II

| Tests | Day 1 | Day 2 | Day 3 | Day 4 | Day 5 |
| --- | --- | --- | --- | --- | --- |
| 1 | 2 vs 10 | 9 vs 3 | 6 vs 4 | 4 vs 8 | 6 vs 2 |
| 2 | 6 vs 4 | 8 vs 4 | 2 vs 6 | 10 vs 6 | 4 vs 10 |
| 3 | 2 vs 6 | 6 vs 10 | 4 vs 8 | 4 vs 6 | 10 vs 8 |
| 4 | 8 vs 2 | 6 vs 8 | 8 vs 2 | 9 vs 6 | 10 vs 6 |
| 5 | 6 vs 9 | 9 vs 6 | 6 vs 10 | 6 vs 2 | 6 vs 9 |
| 6 | 10 vs 6 | 2 vs 6 | 10 vs 8 | 3 vs 9 | 8 vs 2 |
| 7 | 8 vs 4 | 8 vs 10 | 2 vs 10 | 8 vs 6 | 6 vs 8 |
| 8 | 6 vs 8 | 10 vs 4 | 9 vs 3 | 2 vs 10 | 10 vs 2 |
| 9 | 3 vs 9 | 4 vs 6 | 4 vs 10 | 8 vs 10 | 3 vs 9 |
| 10 | 10 vs 4 | 2 vs 10 | 6 vs 9 | 10 vs 4 | 4 vs 6 |
| 11 | 8 vs 10 | 8 vs 2 | 8 vs 6 | 2 vs 8 | 8 vs 4 |
| 12 | 9 vs 6 | 4 vs 8 | 6 vs 10 | 6 vs 2 | 4 vs 10 |
| 13 | 2 vs 8 | 9 vs 3 | 6 vs 2 | 4 vs 8 | 6 vs 4 |
| 14 | 8 vs 10 | 8 vs 2 | 2 vs 8 | 10 vs 8 | 9 vs 6 |
| 15 | 6 vs 2 | 8 vs 10 | 4 vs 10 | 6 vs 10 | 2 vs 10 |
| 16 | 6 vs 10 | 10 vs 4 | 10 vs 2 | 8 vs 2 | 8 vs 10 |
| 17 | 10 vs 2 | 10 vs 6 | 8 vs 6 | 6 vs 8 | 8 vs 4 |
| 18 | 10 vs 4 | 6 vs 8 | 4 vs 8 | 4 vs 6 | 3 vs 9 |
| 19 | 6 vs 8 | 9 vs 6 | 9 vs 3 | 9 vs 6 | 6 vs 2 |
| 20 | 4 vs 6 | 2 vs 10 | 6 vs 4 | 4 vs 10 | 2 vs 8 |
| 21 | 8 vs 4 | 6 vs 2 | 6 vs 9 | 10 vs 2 | 10 vs 6 |
| 22 | 3 vs 9 | 4 vs 6 | 10 vs 8 | 9 vs 3 | 8 vs 6 |

**Supplementary Table S2 (continued).**

Phase III

| Tests | Day 1 | Day 2 | Day 3 | Day 4 | Day 5 |
| --- | --- | --- | --- | --- | --- |
| 1 | 4 vs 7 | 8 vs 3 | 3 vs 8 | 7 vs 4 | 4 vs 9 |
| 2 | 7 vs 6 | 6 vs 7 | 6 vs 3 | 8 vs 9 | 6 vs 7 |
| 3 | 4 vs 9 | 10 vs 5 | 2 vs 9 | 9 vs 10 | 9 vs 7 |
| 4 | 9 vs 8 | 10 vs 9 | 5 vs 10 | 6 vs 3 | 2 vs 9 |
| 5 | 7 vs 9 | 2 vs 9 | 10 vs 9 | 7 vs 10 | 10 vs 7 |
| 6 | 5 vs 10 | 8 vs 9 | 9 vs 8 | 9 vs 7 | 8 vs 3 |
| 7 | 10 vs 9 | 7 vs 4 | 4 vs 7 | 5 vs 10 | 9 vs 10 |
| 8 | 2 vs 9 | 7 vs 9 | 9 vs 7 | 9 vs 2 | 6 vs 3 |
| 9 | 6 vs 3 | 9 vs 4 | 9 vs 4 | 9 vs 4 | 4 vs 7 |
| 10 | 8 vs 3 | 10 vs 7 | 7 vs 10 | 3 vs 8 | 10 vs 5 |
| 11 | 7 vs 10 | 3 vs 6 | 6 vs 7 | 7 vs 6 | 8 vs 9 |
| 12 | 10 vs 5 | 9 vs 7 | 9 vs 2 | 10 vs 5 | 7 vs 6 |
| 13 | 9 vs 2 | 9 vs 2 | 8 vs 9 | 3 vs 6 | 3 vs 6 |
| 14 | 3 vs 6 | 7 vs 10 | 8 vs 3 | 10 vs 9 | 10 vs 9 |
| 15 | 7 vs 4 | 4 vs 9 | 4 vs 9 | 2 vs 9 | 7 vs 4 |
| 16 | 8 vs 9 | 7 vs 6 | 7 vs 4 | 10 vs 7 | 5 vs 10 |
| 17 | 9 vs 7 | 9 vs 10 | 3 vs 6 | 6 vs 7 | 7 vs 9 |
| 18 | 10 vs 7 | 5 vs 10 | 10 vs 7 | 9 vs 8 | 9 vs 4 |
| 19 | 3 vs 8 | 6 vs 3 | 7 vs 6 | 7 vs 9 | 3 vs 8 |
| 20 | 9 vs 4 | 4 vs 7 | 7 vs 9 | 4 vs 7 | 9 vs 8 |
| 21 | 6 vs 7 | 3 vs 8 | 10 vs 5 | 8 vs 3 | 7 vs 10 |
| 22 | 9 vs 10 | 9 vs 8 | 9 vs 10 | 4 vs 9 | 9 vs 2 |

**Supplementary Table S3.** Model selection for the quantitative ability of Asian freshwater turtles in Experiment 1.

| Model | Res. df | Res SS | p |
| --- | --- | --- | --- |
| Full model:  Success = Subject + Ratio + Day + Subject × Ratio + Subject × Day + Ratio × Day + Subject × Ratio × Day + Difference | 104 | 1.1075 |  |
| Full vs. model 1: Subject × Ratio × Day removed  Model 1: Success = Subject + Ratio + Day + Subject × Ratio + Subject × Day + Ratio × Day + Difference | 108 | 1.1319 | 0.68 |
| Model 1 vs. 2: Subject × Ratio removed  Model 2: Success = Subject + Ratio + Day + Subject × Day + Ratio × Day + Difference | 112 | 1.1950 | 0.20 |
| Model 1 vs. 3: Subject × Day removed  Model 3: Success = Subject + Ratio + Day + Subject × Ratio + Ratio × Day + Difference | 112 | 1.1768 | 0.37 |
| Model 1 vs. 4: Ratio × Day removed  Model 4: Success = Subject + Ratio + Day + Subject × Ratio + Subject × Day + Difference | 109 | 1.1402 | 0.38 |
| Model 5: Success = Subject + Ratio + Day + Difference | 117 | 1.2482 |  |
| Model 5 vs. 6: Subject removed  Model 6: Success = Ratio + Day + Difference | 121 | 1.2912 | 0.40 |
| Model 5 vs. 7: Ratio retained  Model 6: Success = Subject + Day + Difference | 118 | 1.3353 | 0.004 |
| Model 5 vs. 7: Day retained  Model 7: Success = Subject + Ratio + Difference | 118 | 1.3211 | 0.03 |
| Model 5 vs. 8: Difference removed  Model 8: Success = Subject + Ratio + Day | 118 | 1.2730 | 0.13 |
| Best-fit model: Success = Ratio + Day |  |  |  |

**Supplementary Table S4.** Model selection for the quantitative ability of Asian freshwater turtles in Experiment 2.

| Model | Res. df | Res SS | p |
| --- | --- | --- | --- |
| Full model  Success = Subject + Ratio + Difference + Subject × Ratio + Subject × Difference + Phase | 143 | 2.4408 |  |
| Full vs. model 1  Model 1: Success = Subject + Ratio + Difference + Subject × Ratio + Phase (Subject × Difference removed) | 147 | 2.4983 | 0.49 |
| Full vs. model 2  Model 2: Success = Subject + Ratio + Difference + Subject × Difference + Phase (Subject × Ratio retained) | 147 | 2.6059 | 0.04 |
| Model 1 vs. 3  Model 3: Success = Subject + Ratio + Difference + Phase (Subject × Ratio removed) | 151 | 2.6366 | 0.09 |
| Model 3 vs. 4  Model 4: Success = Ratio + Difference + Phase (Subject retained) | 155 | 2.8906 | 0.006 |
| Model 3 vs. 5  Model 5: Success = Subject + Difference + Phase (Ratio retained) | 152 | 2.7583 | 0.008 |
| Model 3 vs. 6  Model 6: Success = Subject + Ratio + Phase (Difference removed) | 152 | 2.6393 | 0.69 |
| Model 3 vs. 7  Model 7: Success = Subject + Ratio + Difference (Phase retained) | 153 | 2.8308 | 0.003 |
| Best-fit model: Success = Subject + Ratio + Phase |  |  |  |
